# Suppressed Auger Heating of Hot Carriers in Cu‐Doped Colloidal Quantum Wells

**DOI:** 10.1002/advs.75512

**Published:** 2026-05-03

**Authors:** Junhong Yu, Ke Wang, Yadong Han, Zhenzhong Lian, Songyan Hou, Hilmi Volkan Demir, Manoj Sharma

**Affiliations:** ^1^ College of Physics and Electronic Engineering Chongqing Normal University Chongqing China; ^2^ Guangzhou Institute of Technology Xidian University Guangzhou China; ^3^ LUMINOUS! Centre of Excellence for Semiconductor Lighting and Displays School of Electrical and Electronic Engineering School of Physical and Mathematical Sciences School of Materials Science and Engineering Nanyang Technological University Singapore Singapore; ^4^ Department of Electrical and Electronics Engineering and Department of Physics UNAM‐Institute of Materials Science and Nanotechnology Bilkent University Ankara Turkey; ^5^ Department of Materials Science and Engineering Clayton Campus Monash University Melbourne Victoria Australia

## Abstract

Auger heating represents a major bottleneck for hot carrier (HC) relaxation in colloidal quantum wells (CQWs), delaying carrier accumulation in band‐edge states and diminishing performance in light‐emitting applications. To address this issue, we introduce copper doping in CdSe CQWs to create midgap states, which efficiently suppresses Auger heating without altering their intrinsic structural or optical properties. Ultrafast spectroscopy demonstrates pump‐intensity‐invariant HC cooling dynamics in Cu‐doped CQWs, occurring within ∼0.21 ps at a consistent energy‐loss rate of ∼610 meV/ps, even under high exciton densities (<N> ≈ 4). In contrast, undoped samples exhibit significant cooling deceleration as excitation intensity increases. Combined experimental and theoretical results attribute this ultrafast cooling to rapid hole trapping at Cu^1^^+^ sites, which disrupts the biexcitonic energy‐transfer mechanism responsible for Auger reheating. This work establishes a practical strategy for achieving rapid carrier cooling essential for high‐performance CdSe CQW‐based optoelectronics.

## Graphical abstract

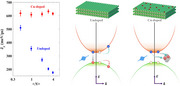

## 1 Introduction

Benefitting from robust excitons at room temperature, narrow emission linewidths with negligible inhomogeneous broadening, and giant absorption cross‐sections, two‐dimensional (2D) zinc‐blende CdSe colloidal quantum wells (CQWs) have emerged as promising materials for light‐emitting applications [1, 2, 3, 4]. For instance, light‐emitting diodes (LEDs) based on CdSe CQWs have recently achieved an external quantum efficiency (EQE) of 26.9% and an operational lifetime (*T*_95_@100 cd/m^2^) exceeding 15 000 h [5, 6]. Moreover, these structures exhibit a giant optical gain coefficient of up to 6600 cm^−^^1^ and the near‐unity quantum efficiency [7], enabling the realization of continuous‐wave pumped lasers [8] and tunable liquid‐state lasers [9]. To further enhance the performance of CdSe CQW‐based devices for practical applications, a detailed understanding of hot carrier (HC) cooling dynamics is essential. This is particularly critical since optically or electrically generated carriers with excess energy represent the initial step in light‐emission processes [10]. Efficient and rapid relaxation of these hot carriers is highly desirable to minimize nonradiative losses to trap states [11, 12, 13].

The cooling dynamics of HCs in semiconductor nanomaterials are governed primarily by electron‐phonon scatterings [13, 14] or the Auger recombination processes [15, 16]. Unlike zero‐dimensional colloidal quantum dots (CQDs), which exhibit discrete electronic states [17], CdSe CQWs possess continuous energy bands, with level spacings significantly smaller than longitudinal optical phonon energies [1, 18]. Consequently, intraband carrier cooling in CQWs does not rely on multi‐phonon emissions, and a phonon bottleneck, common in CQDs, is generally absent [18]. However, previous studies have shown that hot carriers in CdSe CQWs undergo continuous reheating via the biexciton Auger effect [19, 20, 21]. In this process, the recombination energy of one exciton is transferred to another, rather than being emitted as a photon, resulting in the redistribution of excess energy and heating of the electronic system [15]. This Auger reheating mechanism considerably prolongs the hot carrier cooling time, extending it into the picosecond range [18, 19], thereby impeding the quick accumulation of carriers in band‐edge states and limiting performance in light‐emitting applications.

In this work, we demonstrate that copper doping, which introduces midgap states in the host material, effectively suppresses Auger heating of HCs in CdSe CQWs. In contrast to conventional strategies that require significant alterations in morphology, structure, or composition, our approach preserves the intrinsic properties of the CQWs while achieving complete suppression of Auger heating, even at high exciton densities (an average of excitons per CQW, <*N*> ≈ 4). Through femtosecond transient absorption (TA) and ultrafast photoluminescence (PL) spectroscopy, we observe ultrafast, pump‐intensity‐independent HC cooling in Cu‐doped CQWs, occurring within ∼0.21 ps with a nearly constant energy loss rate of ∼610 meV/ps. This stands in stark contrast to the slowed cooling dynamics observed in undoped CQWs, where the cooling time increases from ∼0.25 to ∼0.73 ps as <*N*> rises from 0.49 to 3.89. Combined experimental and theoretical analyses indicate that the rapid capture of holes by Cu^1^^+^ states disrupts the energy‐transfer pathway responsible for Auger heating, thereby enabling the observed intensity‐independent cooling dynamics in Cu‐doped CQWs.

## 2 Results

**FIGURE 1:**
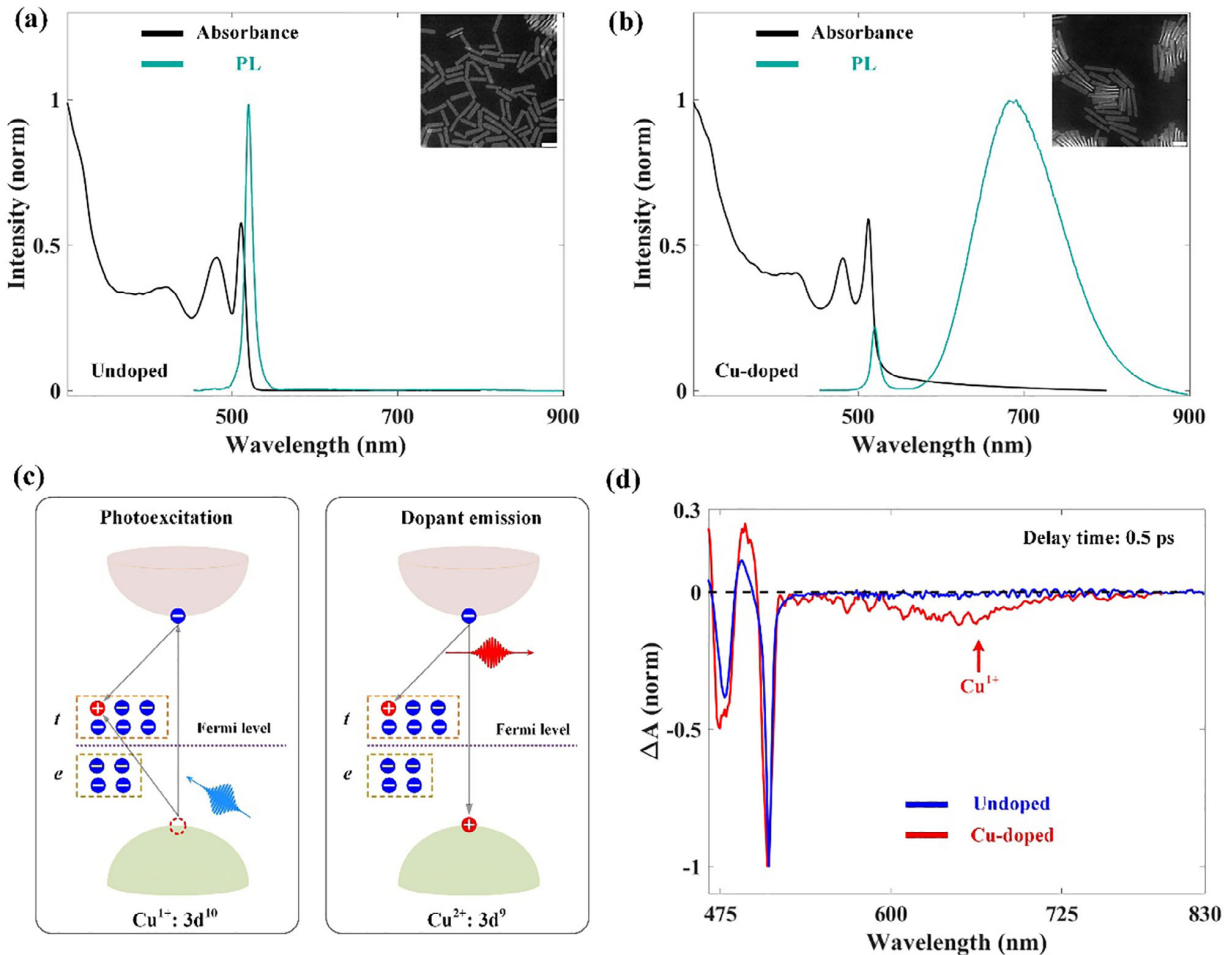
Optical properties of undoped and Cu‐doped 4 ML CdSe CQWs. (a) Optical absorption (blue curve) and PL spectra (red curve) of undoped CQWs. The inset is the TEM image of undoped CQWs with a scale bar of 20 nm. (b) Optical absorption (blue curve) and PL spectra (red curve) of Cu‐doped CQWs (∼70 copper dopants per CQW). The inset is the TEM image of Cu‐doped CQWs with a scalebar of 20 nm. (c) Schematic illustrations of emission mechanisms for Cu‐doped and undoped CQWs. (d) The TA spectrum cutting at 0.5 ps of Cu‐doped and undoped CQWs under the photoexcitation of 460 nm.

Four‐monolayer core‐only undoped and Cu‐doped (∼70 copper dopants per CQW) CdSe CQWs are prepared according to the procedure [16, 22, 23, 24] described in Note S1. Both CQWs are of rectangular shape with the size distributions presented in Figure S1. The absorption spectra of undoped and Cu‐doped CQWs are presented in Figure 1a,b, which shows that the electron/heavy‐hole (HH) and electron/light‐hole (LH) transitions are not affected by copper dopants. Compared to the narrow band edge emission at ∼512 nm in undoped CQWs, Cu‐doped CQWs additionally exhibit a dominant broad emission band centered at ∼700 nm with a bandwidth of ∼180 nm and a Stokes shift as large as ∼670 meV from the electron/HH transition (see Figure 1b). As shown in Figure 1c, Cu ions introduced into CdSe CQW have the (Ar)3d^10^ configuration. These Cu^1+^ oxidation states of predominantly 3d character are conventionally labeled *e* with four‐fold degenerate and *t* with six‐fold degenerate (Cu^1+^: e^4^t^6^) [25, 26]. After photoexcitation, copper dopants quickly localize the photogenerated holes in the valence band (VB) of the host. Cu^1+^ is promoted to Cu^2+^ with the configuration of (Ar)3d^9^ (Cu^1+^: e^4^t^5^), which is therefore activated as a radiative acceptor for electrons in the conduction band (CB). The copper emission process can be expressed as: [Ar]3d^9^ + *e* → [Ar]3d^10^ + *hν*. Meanwhile, the sub‐band‐gap Cu^1+^ to CB transition is also allowed, which manifests in the absorption spectrum as a broad absorption tail on the red side of the HH excitonic feature in Figure 1b. This emission mechanism can be further confirmed by the TA spectra cut at 0.5 ps under 460 nm excitation. As shown in Figure 1d, an additional negative band resonant with the absorption tail is observed in the TA spectrum of Cu‐doped CQWs, indicating that the transition of Cu centres is bleached by band edge pumping and confirming that Cu^1+^ is the essential species to produce the broad copper emission [27].

**FIGURE 2:**
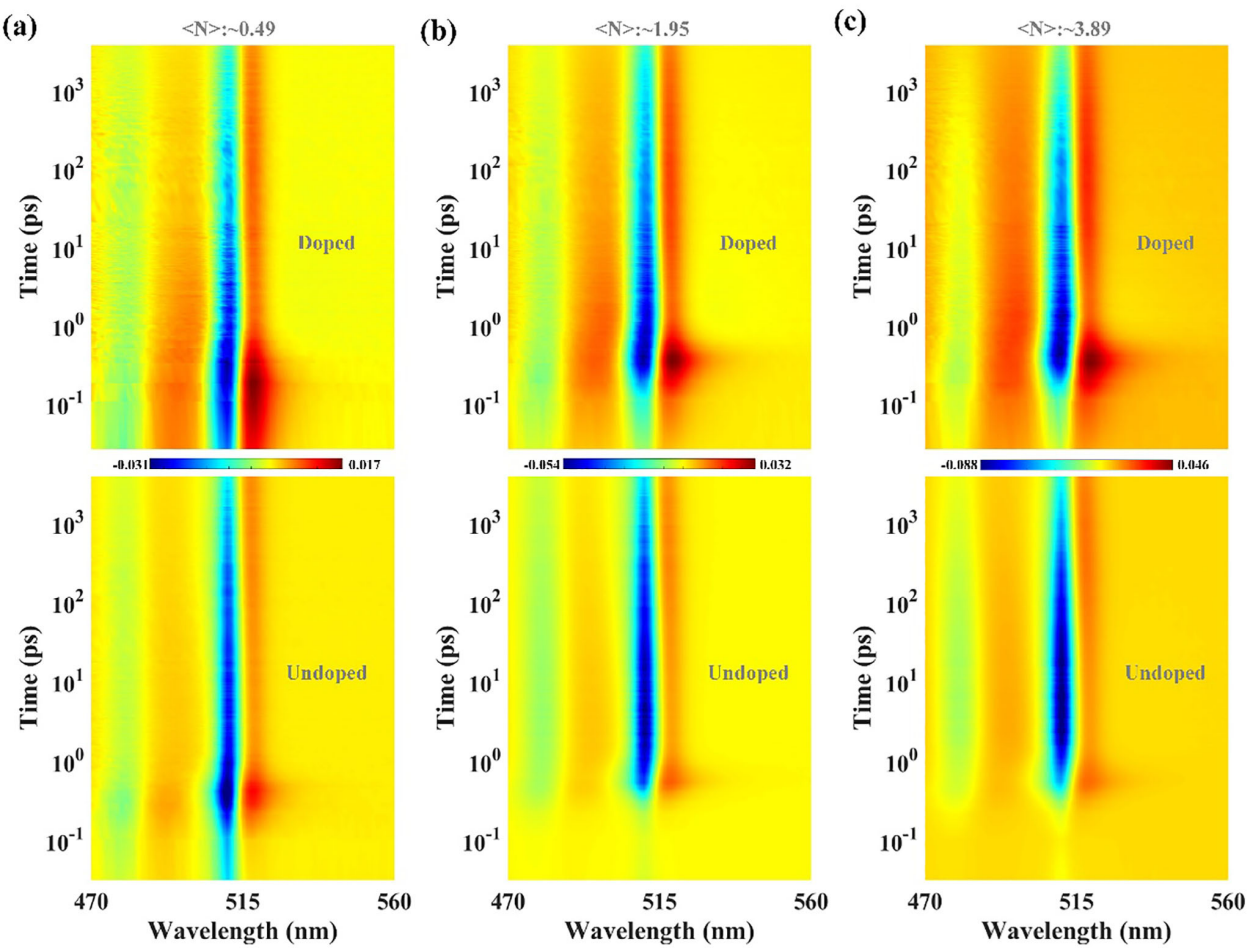
Pseudocolor transient absorption (TA) spectroscopies of Cu‐doped (upper panel) and undoped (lower panel) CQWs for low, intermediate, and high <*N*> values of 0.49 (a), 1.95 (b), and 3.89 (c).

Systematic TA tests are conducted to examine the HC relaxation processes in undoped and copper‐doped CQWs. A 460 nm laser pulse served as the pump source, while a femtosecond supercontinuum probe was used to track the ultrafast dynamics of the photogenerated carriers. The pump fluence is adjusted across a range from 6.8 to 54 µJ/cm^2^, corresponding to an average of excitons (electron−hole pairs) per CQW <*N*> between 0.49 and 3.89. These values are calculated using the relation <*N*> = *σ*×*j* [28, 29], where *σ* represents the absorption cross‐section (3.1 × 10^−14^ cm^−2^) [30] and *j* denotes the incident photon fluence per pulse. Pseudocolor TA maps for the Cu‐doped and undoped CQWs are presented in Figure 2 for low, intermediate, and high <*N*> values of 0.49, 1.95, and 3.89; additional data for other <*N*> values can be found in Figure S2. As shown in Figure 1, the incorporation of copper dopants does not alter the excitonic transition profiles of the host CQWs. Consequently, both samples exhibit two pronounced exciton bleach (XB) signals, corresponding to HH and LH excitons, centered near 482 and 510 nm, respectively.

The TA spectra also display sharp photoinduced absorption (PIA) peaks adjacent to these XB features, a behavior commonly observed in low‐dimensional semiconductors and likely arising from effects such as bandgap renormalization, excitonic linewidth broadening, and biexciton formation. Although the spectral line shapes are similar between undoped and Cu‐doped CQWs, their bleach recovery dynamics reveal clear differences induced by copper doping. In undoped CQWs at <*N*> = 0.49, the recombination follows a single‐exciton decay pattern, as supported by previous studies [31]. With increasing carrier density, a faster recovery component (∼117.4 ps) emerges and gains prominence under higher excitation fluence (Figure S3), consistent with biexciton Auger recombination reported in CdSe CQWs [8, 32, 33, 34]. In contrast, Cu‐doped CQWs exhibit an ultrafast recovery component (∼1.14 ps) even at the lowest excitation level (<*N*> = 0.49, Figure S4). Notably, both the amplitude and lifetime of this fast component remain independent of <*N*> (Figure S5), and its kinetics match the rise of the copper‐related bleach signal at ∼650 nm (Figure S6). This component is therefore assigned to the hole localization process from the valence band of the CQW to the Cu^1^^+^ state, indicating that rapid hole capture quenches biexciton Auger pathways in Cu‐doped CQWs.

**FIGURE 3:**
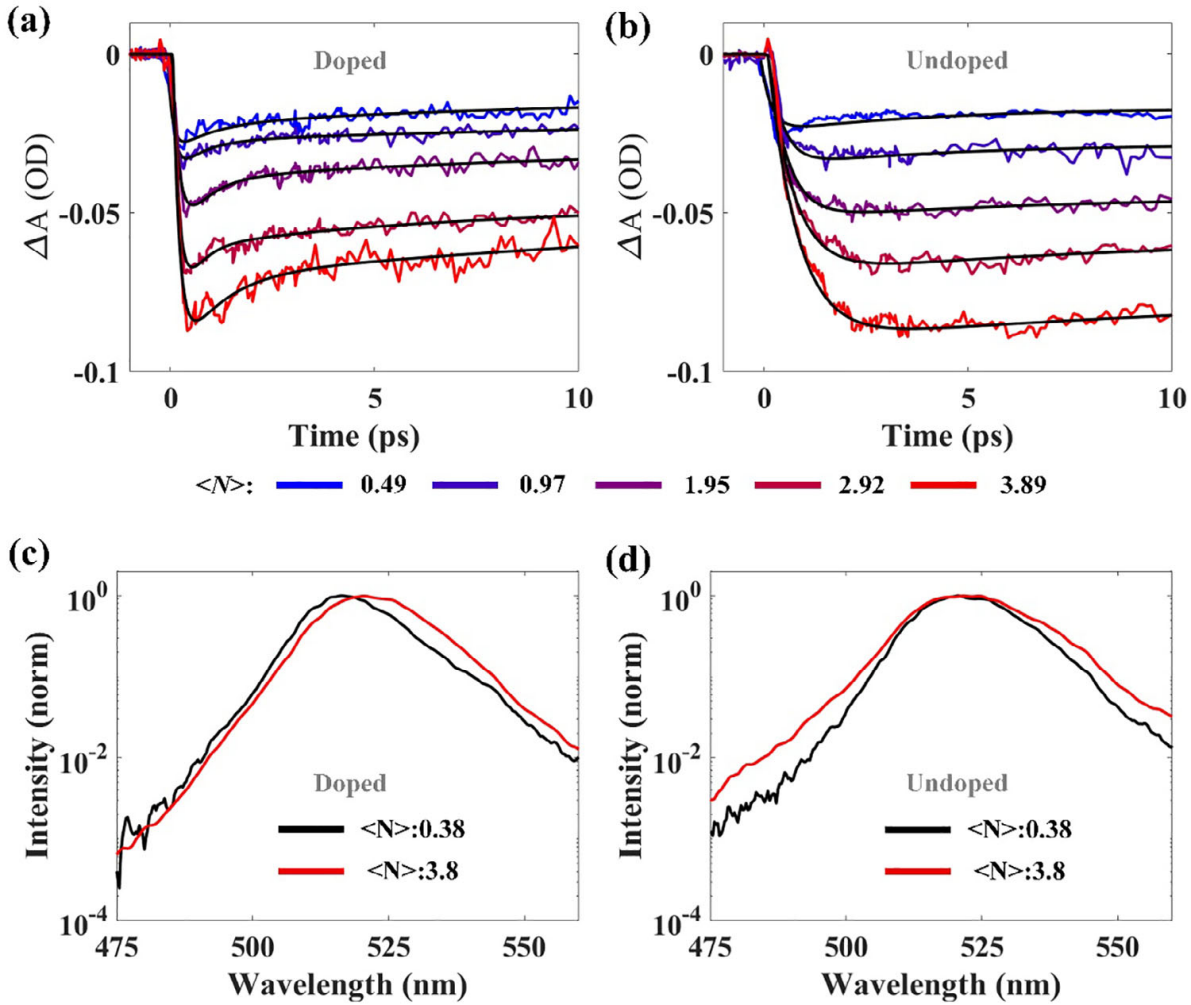
HC dynamics of undoped and Cu‐doped 4 ML CdSe CQWs. (a) The rising edges of TA signals probed at the band edge for Cu‐doped CQWs. (b) The rising edges of TA signals probed at the band edge for undoped CQWs. Solid lines are the global fitting of experimental data. (c) Transient PL spectra of Cu‐doped CQWs with <*N*> = 0.38 (black) and <*N*> = 3.8 (red), which are integrated over 0−50 ps after excitation. (d) Transient PL spectra of undoped CQWs with <*N*> = 0.38 (black) and <*N*> = 3.8 (red), which are integrated over 0−50 ps after excitation.

Owing to the large exciton binding energy and strong quantum confinement in these CQWs, along with spectral overlap between the HH and LH excitonic transitions, no distinct high‐energy tail is observed in the XB band [35]. This makes it infeasible to determine HC temperature via a Boltzmann distribution, an approach commonly applied in studies of weakly confined perovskite nanocrystals [10, 11, 12, 13, 36]. Instead, under above‐bandgap excitation at 460 nm (corresponding to an excess energy of ∼264 meV), we evaluate HC cooling dynamics by tracking the formation dynamics of the XB band, which signals the relaxation of hot carriers to the band edge [10, 35, 37]. To minimize fitting artifacts, the XB signal was averaged over its full width at half maximum (FWHM). As shown in Figure 3b, in undoped CQWs under single‐exciton conditions (<*N*> = 0.49, where the biexciton probability is below 8% as estimated from Poisson statistics) [29], the XB rise time is 249 ± 12 fs. This value is consistent with the theoretical timescale for electron–longitudinal optical (LO) phonon scattering (∼200 fs) in two‐dimensional systems [38], indicating that a phonon bottleneck, often expected from sparse electronic states in strongly confined systems [17], is not observed. This result aligns with earlier reports [18, 19, 20] and can be explained by the continuous density of states in CdSe CQWs. At higher carrier densities (increased <*N*>), the HC cooling lifetime in undoped CQWs is significantly prolonged, reaching 734 ± 9 fs.

To rule out the potential influence of hot phonon bottlenecks, where rapid accumulation of LO phonons under high pump intensity and their reabsorption by carriers slow down the cooling process, on extended HC relaxation, we have performed close‐bandgap excitation (pump wavelength: 500 nm) experiments on undoped CQWs. As illustrated in Figure S7, a similar slowdown in HC cooling is observed at <*N*> = 3.89. Furthermore, a kinetic model incorporating cascaded intraband relaxation and hot phonon reabsorption is developed (see Note S2 and Figure S8). This model predicts a theoretical HC cooling lifetime of ∼270 fs, which diverges considerably from our experimental results. Thus, the delayed HC cooling at high excitation densities is primarily due to Auger heating in undoped CQWs. In marked contrast, HC cooling times in Cu‐doped CQWs under above‐bandgap excitation remain consistently around ∼0.21 ps, showing little variation even at high excitation levels (<*N*> = 3.89, Figure 3a) and suggesting negligible Auger heating effects. Please note that in the single‐exciton regime (<*N*> = 0.49), Cu‐doped CQWs exhibit a similar but slightly faster cooling time (∼210 fs) compared to undoped CQWs (249 ± 12 fs). This minor variation arises because Poisson statistics dictate a small probability (< 8%) of biexciton generation at this fluence, introducing residual Auger heating in undoped CQWs. Furthermore, the ultrafast hole capture in Cu‐doped CQWs completely decouples the electron, eliminating hole‐related interference and allowing for undisturbed electron‐phonon relaxation. Similarly, rapid cooling is also seen in Cu‐doped CQWs under close‐bandgap excitation (Figure S9). Furthermore, Auger heating is a delayed reheating process driven by multi‐exciton Auger recombination, which typically occurs on a timescale of several picoseconds in CdSe CQWs. Since the holes are strongly localized at the Cu^1+^ states within ∼1.14 ps, the continuous reheating cycle is aborted, allowing the electrons to cool undisturbed at their intrinsic LO‐phonon scattering rate (∼0.21 fs).

To further corroborate the suppression of Auger heating in Cu‐doped CQWs, we conducted transient photoluminescence (PL) measurements integrated over 0–10 ps (Figure 3c,d). The undoped CQWs exhibit noticeable broadening on the high‐energy side of the PL spectrum as <*N*> increases. As previously reported by Pelton et al. [19, 20], this high‐energy tail arises from radiative recombination of hot carriers and reflects the contributions of Auger heating. Conversely, at high excitation fluences (<*N*> = 3.8), the transient PL spectrum of Cu‐doped CQWs exhibits only a minor, symmetric broadening (Figure 3c) resulting from typical exciton‐exciton scattering, which fundamentally differs from the pronounced, asymmetric high‐energy tail observed in undoped CQWs (Figure 3d). Accordingly, the absence of this asymmetric tail in Cu‐doped CQWs confirms that the photogenerated carriers do not undergo continuous reheating cycles, demonstrating the effective suppression of Auger heating. Furthermore, we have extracted the temporal evolution of the HC temperatures (*T*_c_) for both undoped and Cu‐doped CQWs with <*N*> = 3.8 by fitting the high‐energy tail of the transient PL spectra to a Maxwell‐Boltzmann distribution [13, 14] (see Figure S10). For undoped CQWs, the experimental data can only be accurately fitted by explicitly including an Auger heating term, confirming that multi‐exciton recombination continuously reheats the carrier population. Conversely, *T*_c_ in Cu‐doped CQWs drops to the lattice temperature within 300 fs without any sustained high‐temperature plateau.

**FIGURE 4:**
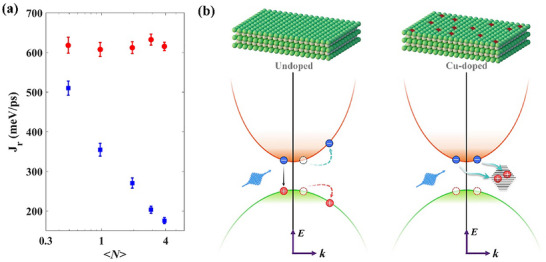
Analysis of the energy loss rate of HCs. (a) The evolution of energy loss rates (J_r_) with <*N*> for undoped (blue dots) and Cu‐doped (red dots) CQWs. (b) Schematic representation of different HC cooling scenarios or undoped and Cu‐doped CQWs.

By dividing the initial excess energy (Δ*E*) by the characteristic HC cooling lifetime (*τ_hc_*), we obtain an estimate of the average energy loss rate (J_r_) as J_r_ = Δ*E*/(2*τ_hc_*) [35, 38], where Δ*E* = *E*_exc_ − *E*_g_. The resulting <*N*>‐dependent values of J_r_ are shown in Figure 4a. At low excitation densities (<*N*> < 1), the energy loss rates for both samples fall within the range of 0.5–0.7 eV/ps, consistent with values reported in other semiconductor quantum wells where neither hot phonon bottlenecks nor Auger heating play a significant role [35, 39, 40]. As <*N*> increases from ∼0.49 to ∼3.89, J_r_ for undoped CQWs decreases markedly by several times, reaffirming that relaxed carriers are re‐excited to higher energies via Auger heating. In contrast, Cu‐doped CQWs exhibit a large and nearly constant J_r_ across all tested <*N*> values, providing clear evidence of suppressed Auger heating. A schematic illustration of the Auger heating contributions is presented in Figure 4b. If multiple excitons are excited simultaneously in undoped CQWs, the intrinsic energy from exciton recombination via the Auger effect is recycled back into the system as kinetic energy, reheating carriers, and leading to a dramatic slowdown of the overall cooling dynamics.​ It is worth mentioning that compared to the fundamental Auger recombination process, Auger heating is a collective and positive feedback mechanism where the energy from multiple Auger recombination events is converted into thermal energy within the carrier population (i.e., heating of the whole electronic system) [15], leading to a sustained increase in the average carrier temperature and a dramatic slowdown of the overall cooling process. This could partially explain why *τ_Auger_* is much longer than *τ_hc_* in CQWs, but still can dramatically decelerate the HC cooling process. Notably, Auger heating is effectively suppressed in Cu‐doped CQWs due to efficient hole localization. The strongly localized hole states substantially reduce wavefunction overlap among carriers [41, 42, 43], lowering the probability of three‐body collisions and effectively blocking the energy‐transfer pathway required for Auger heating. This could also explain the different hot carrier cooling lifetime in Cu‐doped CQDs and CQWs. In CQDs, discrete electronic states and large energy gaps establish a natural phonon bottleneck. When Cu‐doping decouples the electron and hole to shut down the Auger cooling pathway, electrons become trapped, resulting in long‐lived hot carriers. In stark contrast, 2D CdSe CQWs possess continuous energy bands, inherently avoiding this phonon bottleneck. Consequently, with the dominant Auger heating effect suppressed by Cu dopants, hot carriers can simply rely on efficient electron‐LO phonon scattering to rapidly relax to the band edge within ∼210 fs.

## 3 Conclusion

In summary, we have developed a general and efficient approach for overcoming Auger heating in CdSe CQWs through strategic copper doping. This method successfully eliminates the carrier cooling bottleneck while fully retaining the advantageous intrinsic properties of the quantum wells. The introduced midgap states serve as efficient hole traps, abruptly terminating the Auger energy‐transfer cycle and resulting in excitation‐independent ultrafast cooling. This fundamental insight not only resolves a long‐standing cooling limitation in CQWs but also opens a viable pathway for enhancing various light‐emitting devices. Our work underscores the potential of dopant‐host interactions for tailoring carrier dynamics in nanoscale semiconductors, providing a foundation for future high‐performance optoelectronics operating under high exciton densities.

## Conflicts of Interest

The authors declare no conflicts of interest.

## Supporting information




**Supporting File**: advs75512‐sup‐0001‐SuppMat.docx.

## Data Availability

The data that support the findings of this study are available from the corresponding author upon reasonable request.
