# Supplementary material for: Suppressed Auger Heating of Hot Carriers in Cu‐Doped Colloidal Quantum Wells
Source: Adv Sci (Weinh). 2026 May 3;13(42):e75512. doi: 10.1002/advs.75512 (PMC13335709; doi:10.1002/advs.75512)
Supplement: Supplementary file 1 — Supporting File: advs75512‐sup‐0001‐SuppMat.docx. [file ADVS-13-e75512-s001.docx]

**SUPPLEMENTARY INFORMATION**

**Suppressed Auger Heating of Hot Carriers in Cu-doped Colloidal Quantum Wells**

Junhong Yu,^1,*^ Ke Wang,^1^ Yadong Han,^1^ Zhenzhong Lian,^1^ Songyan Hou,^2^

Hilmi Volkan Demir,^3,4,*^ Manoj Sharma,^5,*^

*^1^College of Physics and Electronic Engineering, Chongqing Normal University, Chongqing, 401331, China*

*^2^Guangzhou Institute of Technology, Xidian University, Guangzhou 510555, China*

*^3^LUMINOUS! Centre of Excellence for Semiconductor Lighting and Displays, School of Electrical and Electronic Engineering, School of Physical and Mathematical Sciences, School of Materials Science and Engineering, Nanyang Technological University, Singapore 639798, Singapore*

*^4^Department of Electrical and Electronics Engineering and Department of Physics, UNAM-Institute of Materials Science and Nanotechnology, Bilkent University, Ankara, Turkey*

*^5^Department of Materials Science and Engineering, Monash University, Clayton Campus, Melbourne, Victoria 3800, Australia*

^*^*To whom correspondence should be addressed. Email: jyu012@e.ntu.edu.sg (JY); HVDEMIR@ntu.edu.sg (HVD); manoj.sharma@monash.edu (MS).*

# Supplementary Note 1: The ICP-MS measurements

The doping concentration is determined by the Inductively Coupled Plasma Mass Spectrometry (ICP-MS) measurements. Specifically, 50 μL of different doped CQWs solutions were cleaned 3 times with excess ethanol to remove excess cadmium or surface-adsorbed copper ions. After that, the solution of doped CQWs is precipitated with excess ethanol, and the resulting precipitate is dissolved with 2% of 65% HNO3 to digest the CQWs for ICP-MS measurements. The average dimensions of these 4 ML copper-doped CdSe CQWs measured by TEM microscopy are (38.9 ± 3.9) × (5.8 ± 0.9) × 1.2 nm³, which suggests ~5,600 cadmium and ~4,400 selenium atoms in one CQW. Using ICP-MS measurements, the Cu content was determined as Cu/(Cu + Cd) = 0.10, 0.27, 0.43, 0.57, and 1.2%, which correspond to approximately ~5, ~15, ~24, ~32, and ~70 Cu atoms per CQW, respectively.

# Supplementary Note 2: Material synthesis

Cadmium myristate was synthesized following a previously reported procedure. In a typical preparation, 1.23 g of cadmium nitrate tetrahydrate was dissolved in 40 mL of methanol, while 3.13 g of sodium myristate was separately dissolved in 250 mL of methanol under vigorous stirring. The two solutions were then mixed and allowed to stir for about 1 hour. The resulting whitish product was collected by centrifugation, and the precipitate was redispersed in methanol. This washing process was repeated three to four times to remove any unreacted precursors. Finally, the purified white precipitate was dried under vacuum for 24 hours and stored in a desiccator for further use.

Copper dopant precursor was prepared by adding 30 mg of copper (II) acetate in 3.6 mL of tri-octyl phosphine, and 0.4 mL ODE in a N_2_-filled glove box. The mixture was stirred at 90 °C for 8 h and stored in a glove box for further use. The atomic percentages of copper dopant ions in the doped CQWs host are estimated using Inductively Coupled Plasma Mass Spectrometry (ICP-MS) analysis.

# Supplementary Note 2: TA measurements

Transient absorption spectroscopy: A 1 kHz regenerative amplifier with an 800 nm output wavelength and a 100 fs pulse width, which is seeded by the 80 MHz mode-locked Ti-sapphire oscillators, was used to produce the pump pulse with a maximum energy of mJ. The pump pulse then went through the optical parametric amplifier (OPA) to produce the desired excitation energy for fs-TA experiments. To generate the white light continuum probe beam spanning from 400 to 1,500 nm, we have split a small portion of the fundamental 800 nm pulse from the regenerative amplifier with an energy of ~10 μJ and then incident on sapphire crystals with different thicknesses for the continuum probe beam generation. A commercial spectrometer was used to collect the probe beam spectra in the ultraviolet-visible region with and without the pump excitations.

# Supplementary Note 3: trPL measurements

Time-resolved PL (trPL) measurements are performed with a streak camera. The 400-nm pump laser pulses for trPL are generated from a 1000 Hz regenerative amplifier. The beam from the regenerative amplifier has a center wavelength at 800 nm, a pulse width of around 150 fs and is seeded by a mode-locked Ti-sapphire oscillator. 400-nm pump laser was obtained by frequency doubling the 800-nm fundamental regenerative amplifier output using a BBO crystal. All measurements are performed in the solid film at room temperature in ambient air (53±2% humidity) conditions.

# Supplementary Figure 1

**
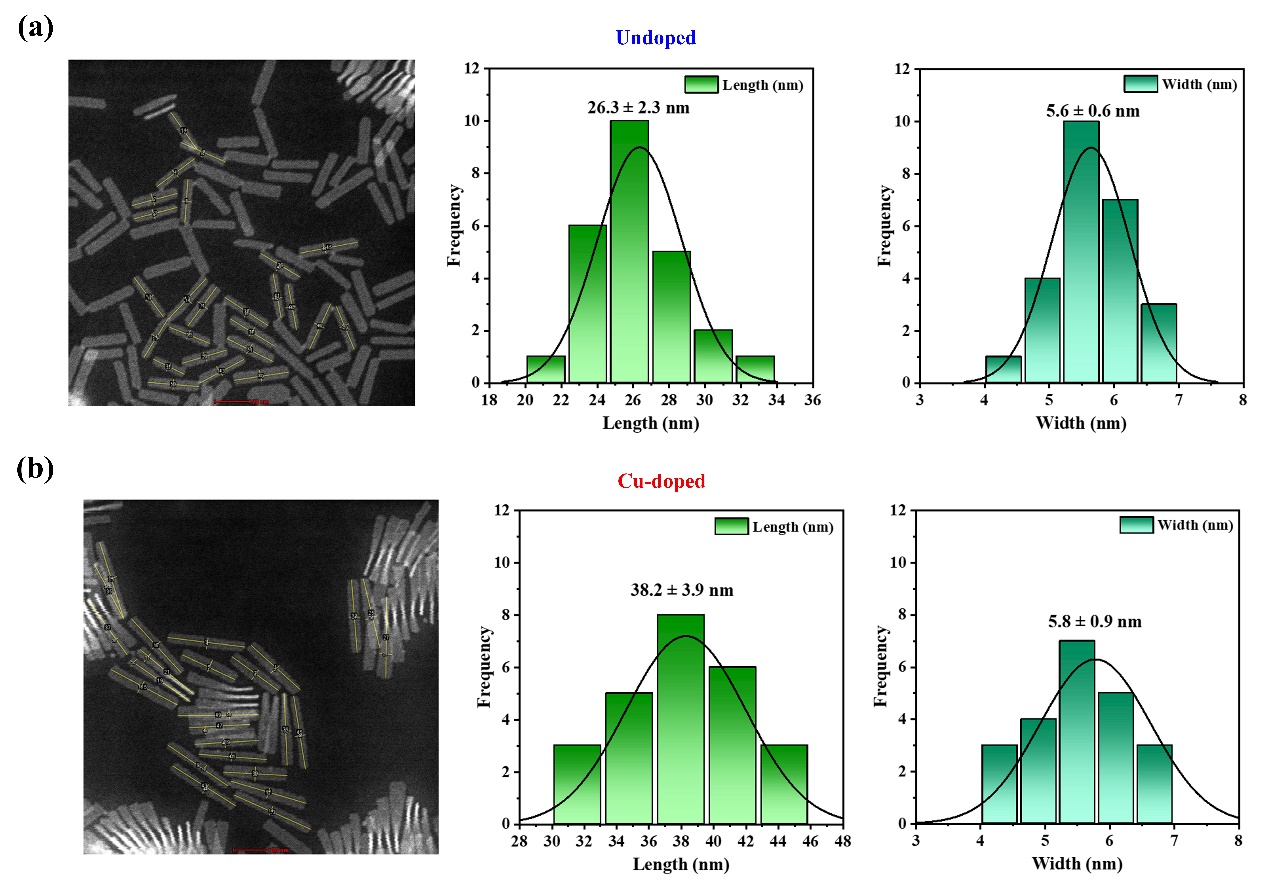
**

**Figure S1. The size distributions of undoped and Cu-doped CQWs.**

# Supplementary Figure 2


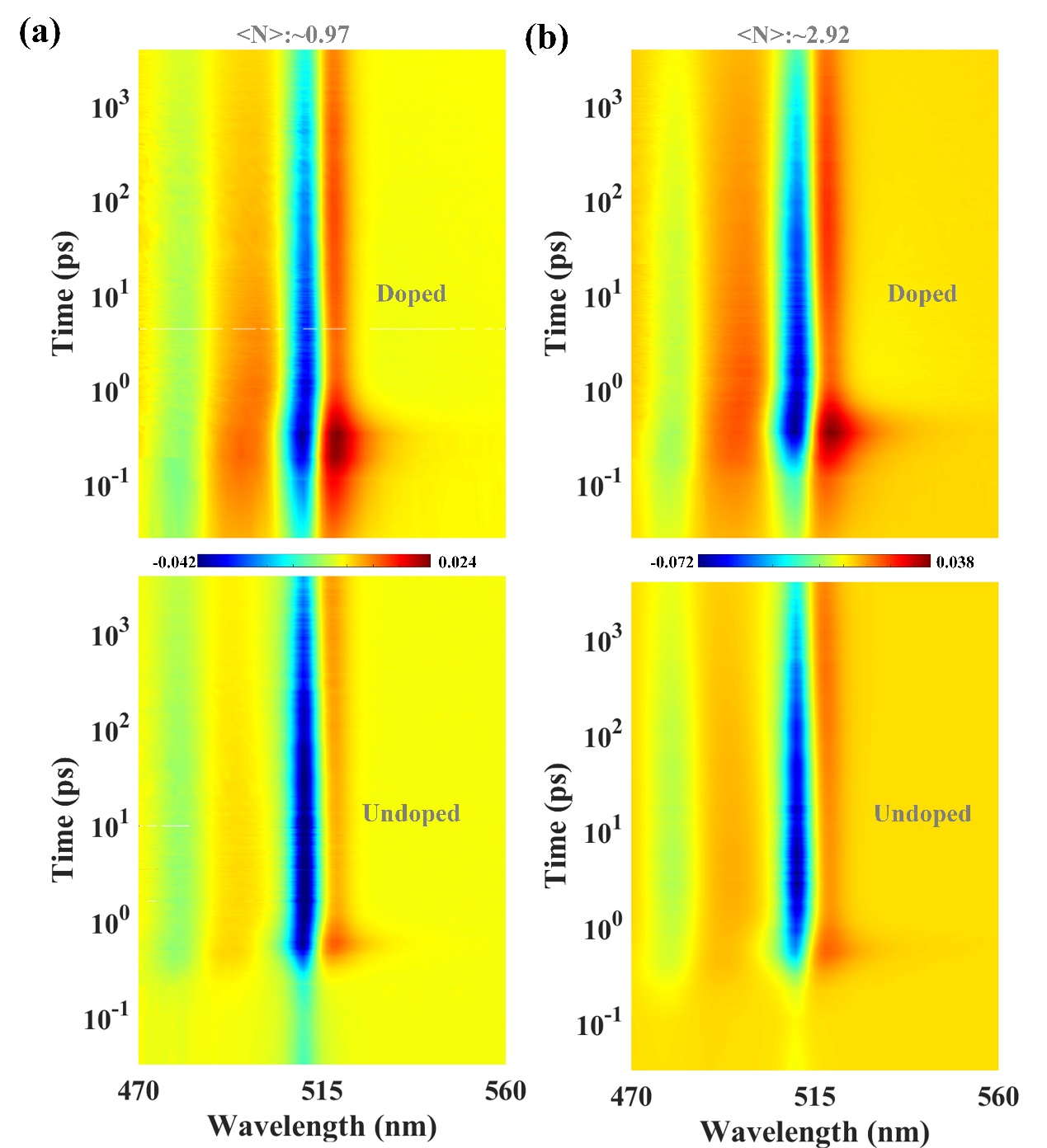


**Figure S2. Pseudocolor transient absorption (TA) spectroscopies of Cu-doped (upper panel) and undoped (lower panel) CQWs for low, intermediate, and high <*N*> values of 0.97 (a) and 2.92 (b).**

# Supplementary Figure 3


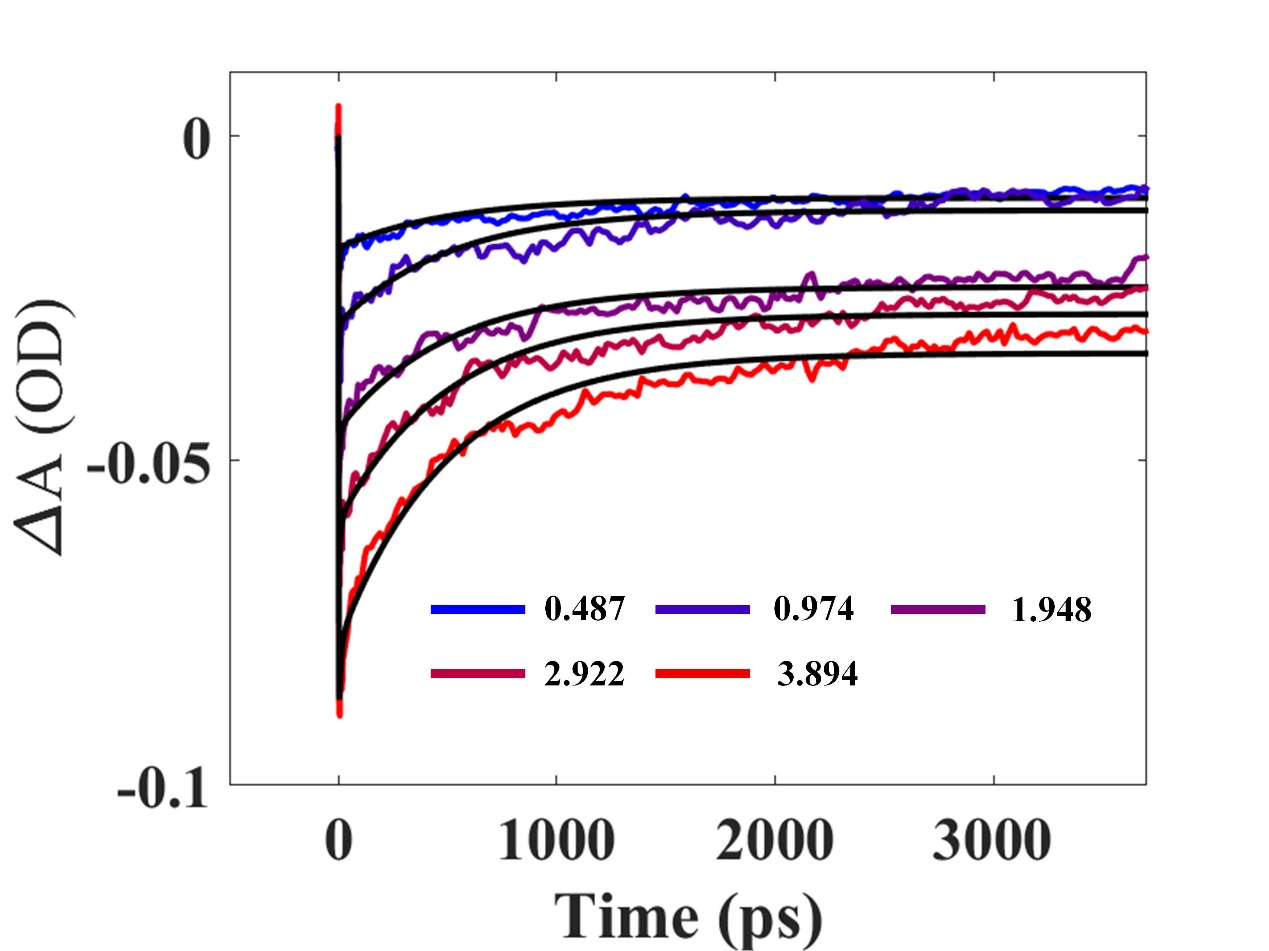


**Figure S3. The TA kinetics of undoped CdSe CQWs probed at the heavy-hole/electron transition as a function of different *<N>* values. The excitation wavelength is 460 nm.**

# Supplementary Figure 4


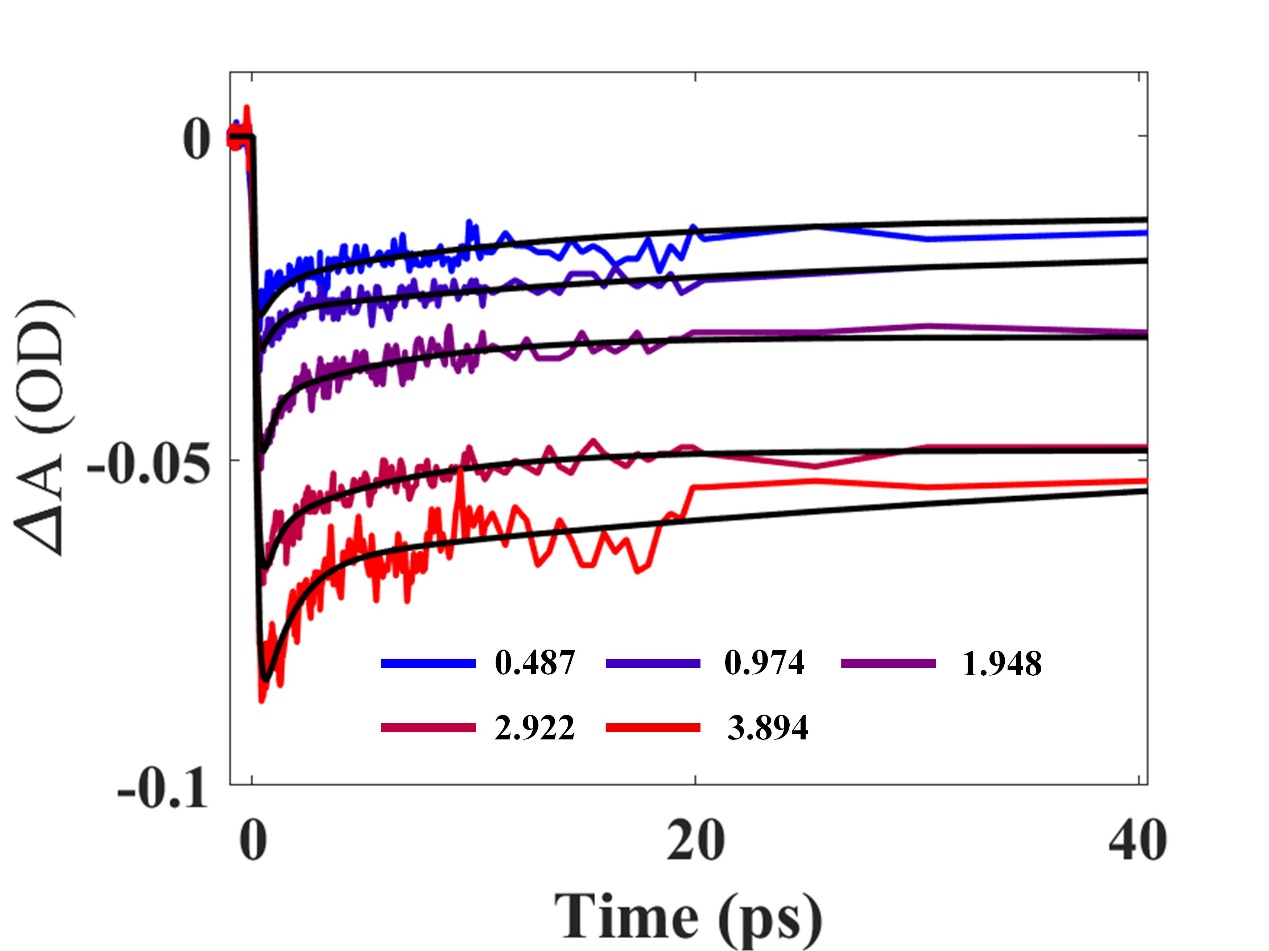


**Figure S4. TA kinetics of Cu-doped CdSe CQWs probed at the heavy-hole/electron transition as a function of different *<N>* values. The excitation wavelength is 460 nm.**

# Supplementary Figure 5


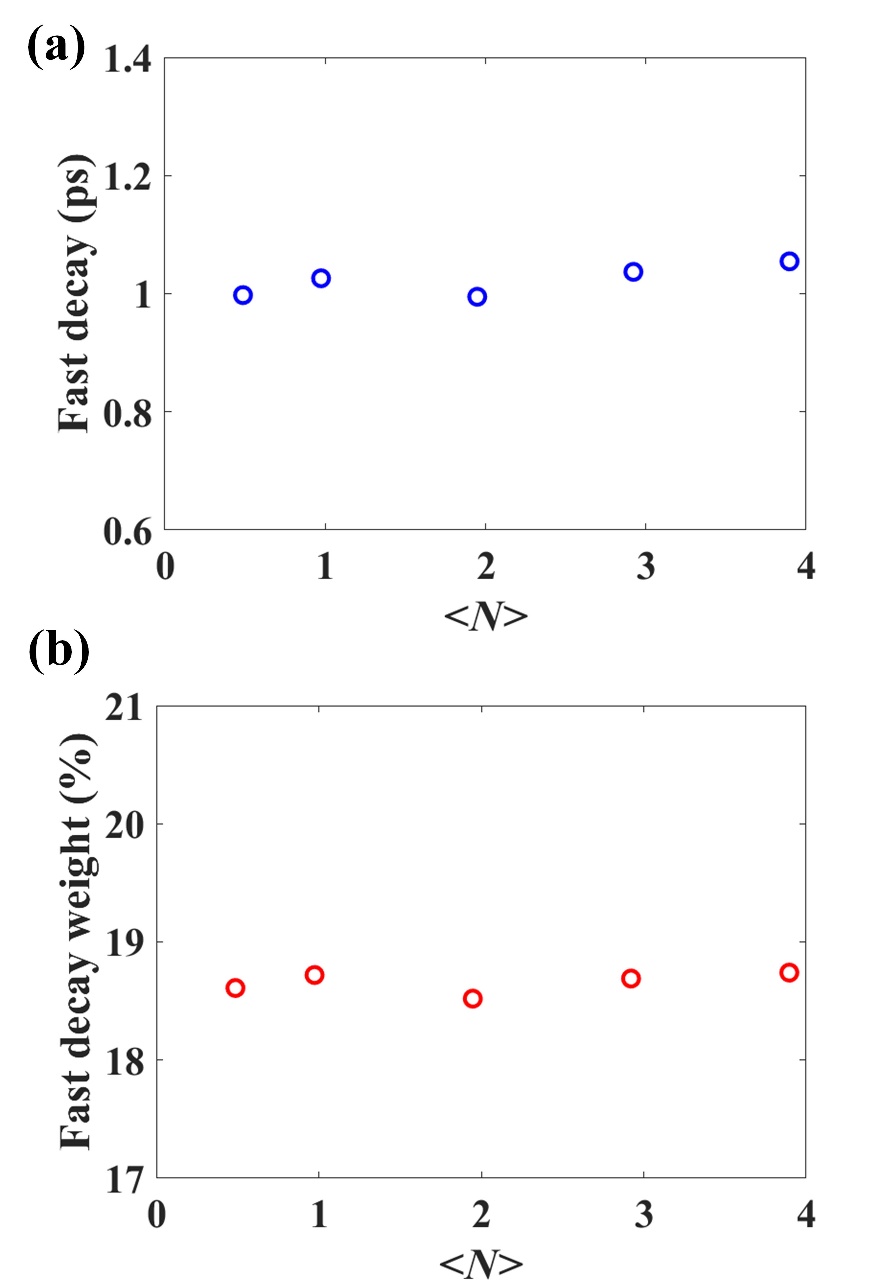


**Figure S5. Lifetime (a) and amplitude (b) of the fast recovery component in Fig. S3 as a function of different *<N>* values.**

# Supplementary Figure 6


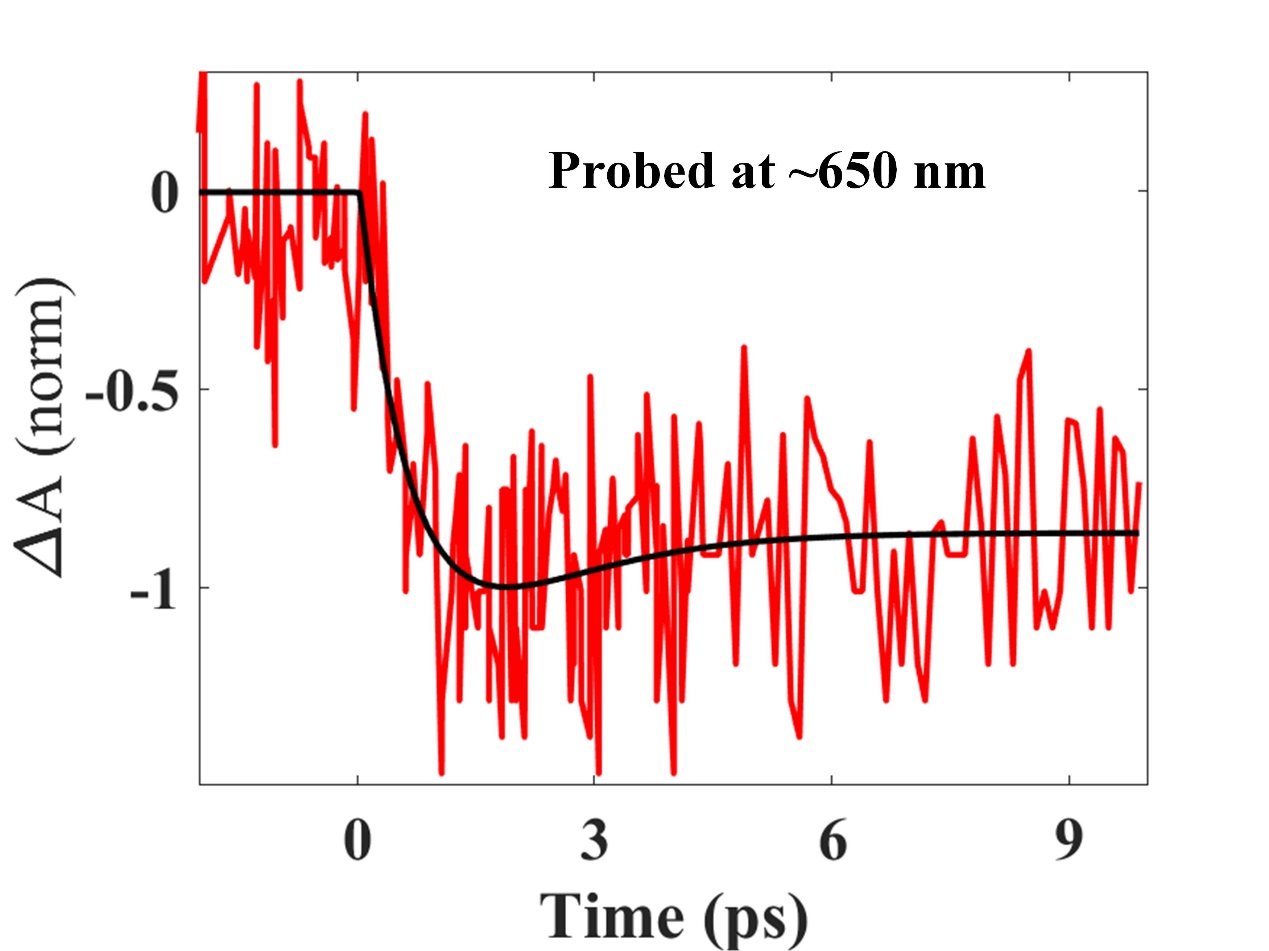


**Figure S6. Copper-related TA signal probed at the copper-induced bleaching band (see Fig. 1). The excitation wavelength is 460 nm.**

# Supplementary Figure 7


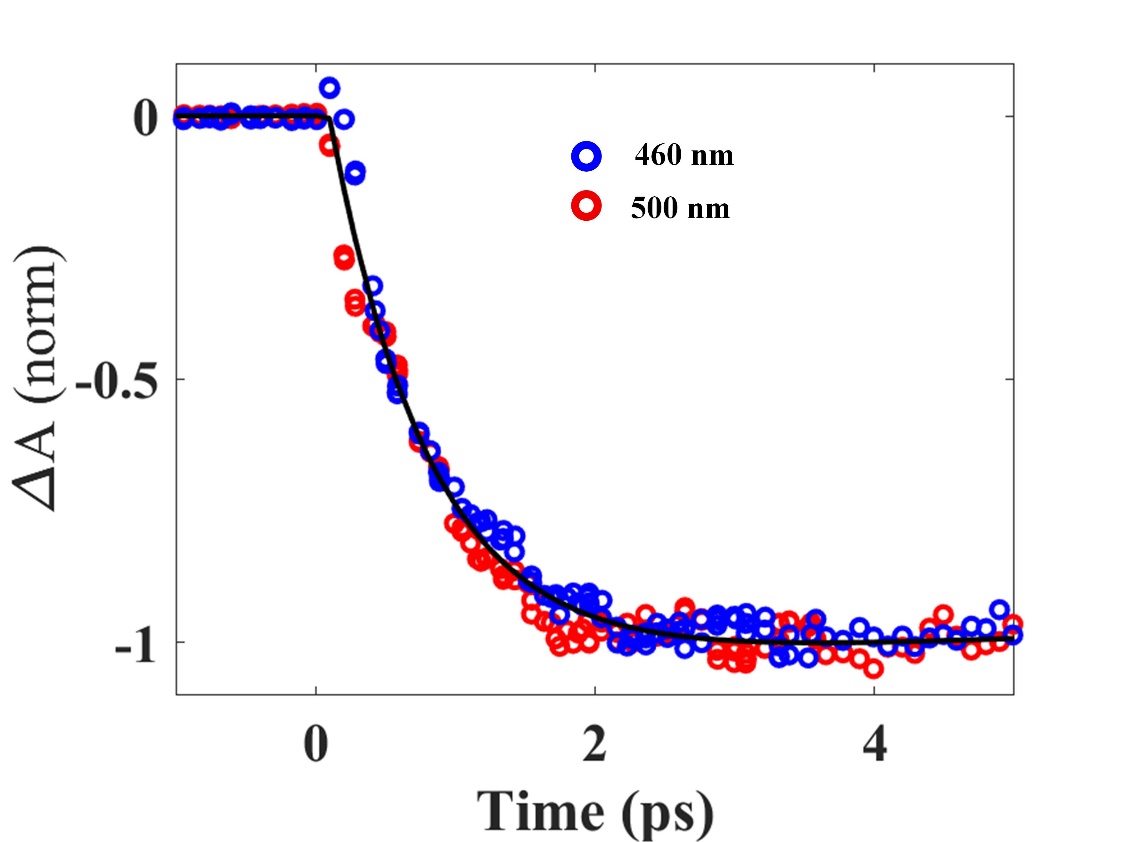


**Figure S7. XB dynamics of undoped CQWs with two different excitation wavelengths. The <*N*> value for both cases is around 3.89. The black curve is the global fitting of experimental data.**

# Supplementary Note 4

With the excessive energy of ~264 meV (i.e., pump wavelength: 460 nm) and the LO phonon energy of ~26 meV in CdSe CQWs, we can approximately treat the HC phonon cooling dynamics by using a ten-step cascaded intraband relaxation process (i.e., for each step, the HC scatter with the lattice and emit one LO phonon). To mimic the reabsorption of LO phonons in the HC cooling process, we have assumed that HCs have the probability of being reheated by LO phonons at each cooling step. Accordingly, a modified rate equation model^[1-3]^ is adapted to describe the hot carrier-phonon interaction and the hot carrier cooling process in undoped CdSe CQWs:

$$\begin{matrix} \frac{dN_{10}}{dt}=G(t)-\frac{N_{10}N_{phonon}}{\tau_{\text{hc}}}+\frac{N_{9}N_{phonon}}{\tau_{e-ph}} \\ \frac{dN_{9}}{dt}=\frac{N_{10}N_{phonon}}{\tau_{\text{hc}}}-\frac{N_{9}N_{phonon}}{\tau_{e-ph}}-\frac{N_{9}N_{phonon}}{\tau_{\text{hc}}}+\frac{N_{8}N_{phonon}}{\tau_{e-ph}} \\ \frac{dN_{8}}{dt}=\frac{N_{9}N_{phonon}}{\tau_{\text{hc}}}-\frac{N_{8}N_{phonon}}{\tau_{e-ph}}-\frac{N_{8}N_{phonon}}{\tau_{\text{hc}}}+\frac{N_{7}N_{phonon}}{\tau_{e-ph}} \end{matrix}$$

……………..

$$\begin{matrix} \frac{dN_{2}}{dt}=\frac{N_{3}N_{phonon}}{\tau_{\text{hc}}}-\frac{N_{2}N_{phonon}}{\tau_{e-ph}}-\frac{N_{2}N_{phonon}}{\tau_{\text{hc}}}+\frac{N_{1}N_{phonon}}{\tau_{e-ph}} \\ \frac{dN_{1}}{dt}=\frac{N_{2}N_{phonon}}{\tau_{\text{hc}}}-\frac{N_{1}N_{phonon}}{\tau_{e-ph}}-\frac{N_{1}N_{phonon}}{\tau_{\text{hc}}}+\frac{N_{0}N_{phonon}}{\tau_{e-ph}} \\ \frac{dN_{0}}{dt}=\frac{N_{1}N_{phonon}}{\tau_{\text{hc}}}-\frac{N_{0}N_{phonon}}{\tau_{e-ph}} \end{matrix}$$

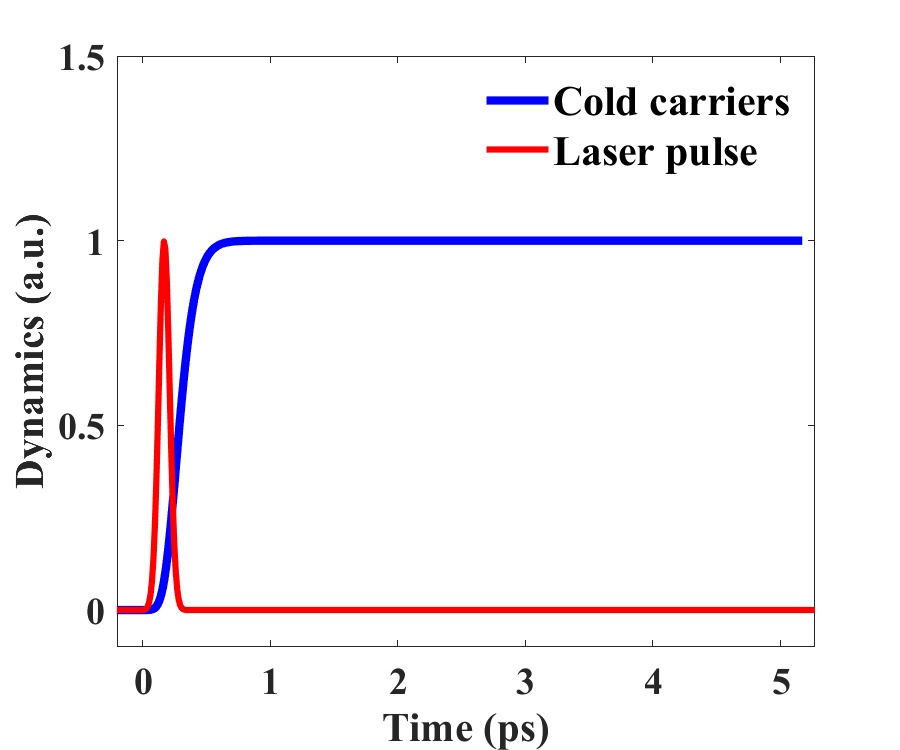


**Figure S8. The simulated carrier cooling dynamics considering the hot phonon bottlenecks.**

where *N_i_* is the population of carriers at each cooling step (e.g., *N*_0_ represents the cold carriers at the band edge and *N*_10_ represents the hot carriers with the excessive energy of ~264 meV), *N*_phonon_ is the population of LO phonons, *G(t)* represents the excitation source, and *τ_hc_* is the hot carrier cooling lifetime at each step (249/10 ≈ 25 fs based on the experimental data in Fig. 3). *τ_e-ph_* is the scattering timescale (~200 fs) of electrons with longitudinal optical (LO) phonons in two-dimensional systems. Based on the rising dynamics of *N*_0_ in **Fig. S8**, we have determined the HC cooling lifetime with the contribution of hot phonon bottleneck is ~270 fs, which is much faster than the experimental value.

# Supplementary Figure 9


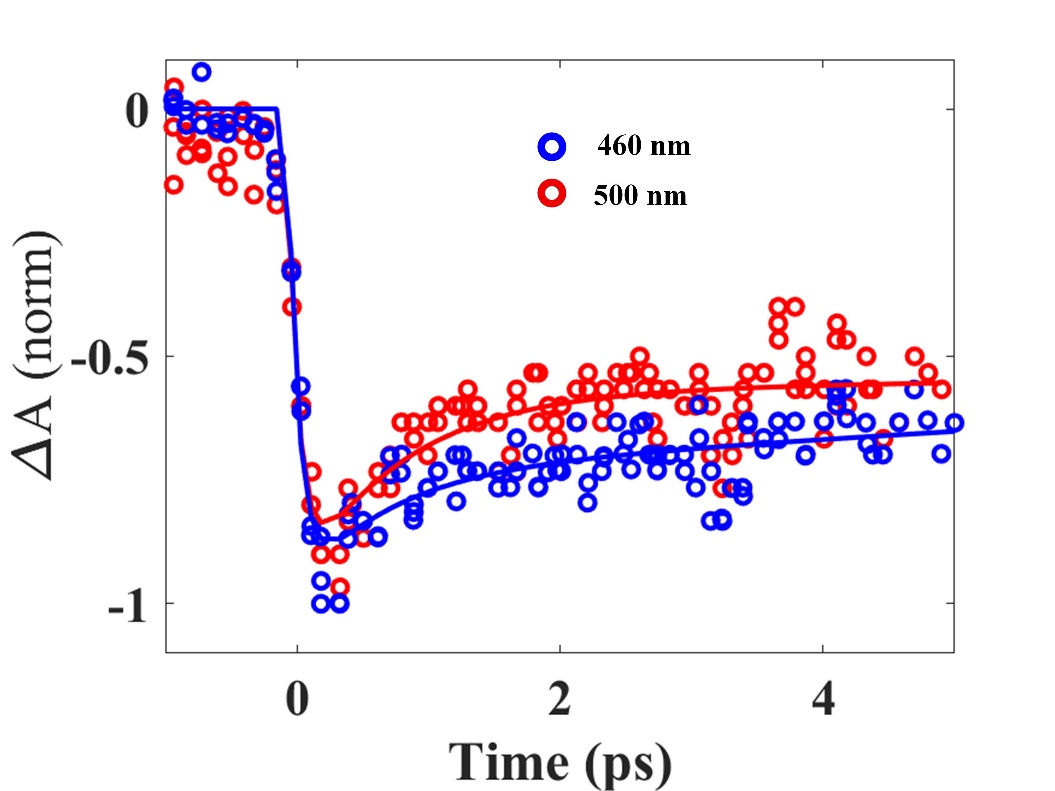


**Figure S9. XB dynamics of Cu-doped CQWs with two different excitation wavelengths. The <*N*> value for both cases is around 3.89. The solid lines are the global fitting of experimental data.**

# Supplementary Figure 10


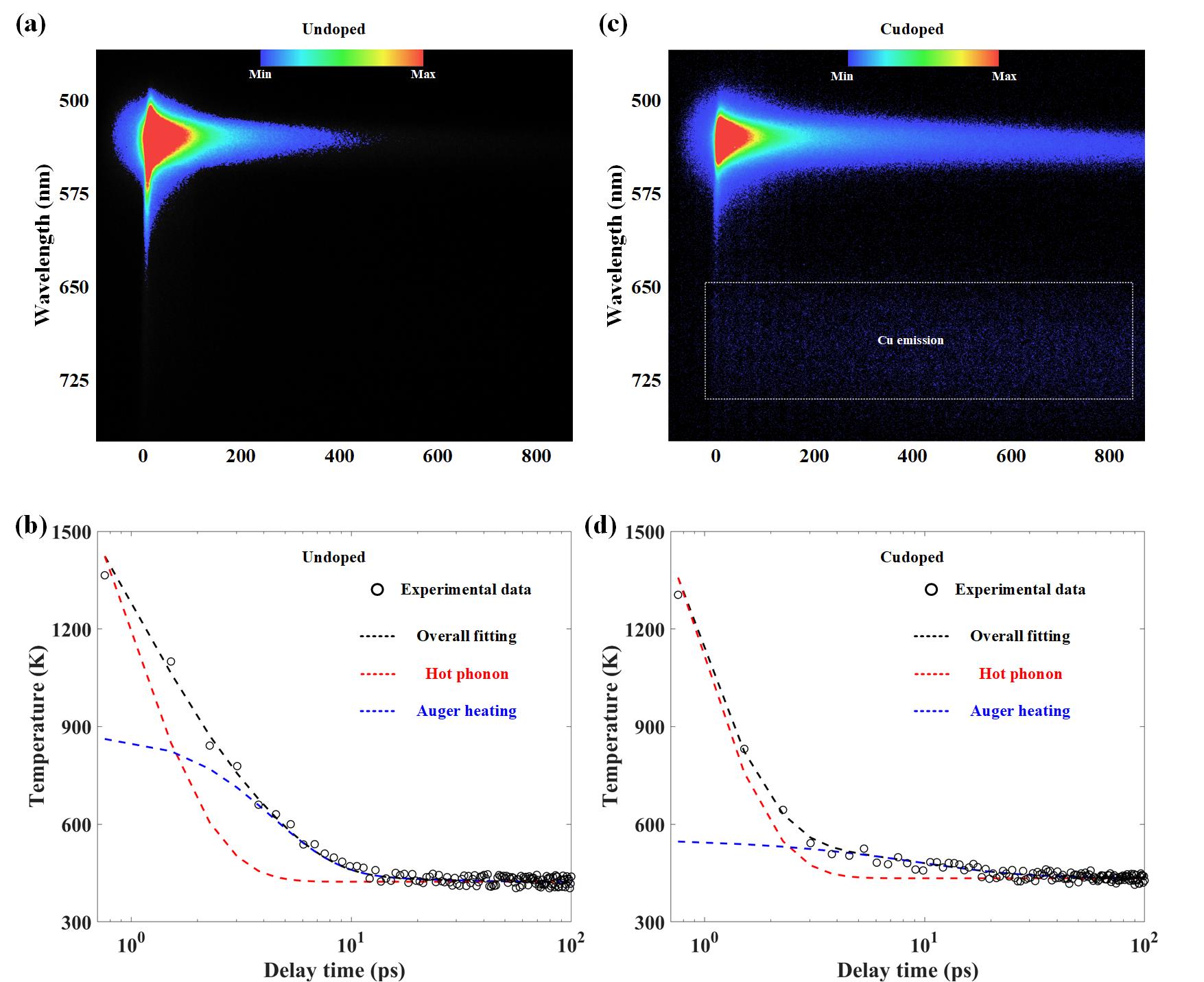


**Figure S10.**  **The time-resolved evolution of the transient PL spectra. (a) The 2D streak camera image of undoped CQWs with <*N*> = 3.8. (b) Analysis of Hot carrier cooling dynamics of undoped CQWs. Black circles: hot carrier temperature extracted from the transient PL spectra. The lines show the fitted HC cooling dynamics with hot-phonon effect only, with Auger heating effects only, and with both effects. (c) The 2D streak camera image of doped CQWs with <*N*> = 3.8. (d) Analysis of Hot carrier cooling dynamics of doped CQWs. Black circles: hot carrier temperature extracted from the transient PL spectra. The lines show the fitted HC cooling dynamics with hot-phonon effect only, with Auger heating effects only, and with both effects.**

# References

1. Yu, J.; Sharma, M.; Delikanli, S.; Birowosuto, M. D.; Demir, H. V.; Dang, C. Mutual Energy Transfer in a Binary Colloidal Quantum Well Complex. *J. Phys. Chem. Lett.* **2019**, 10, 5193−5199.

2. Carneiro, L. M. et al. Excitation-wavelength-dependent small polaron trapping of photoexcited carriers in *alpha*-Fe_2_O_3_. *Nat. Mater.* **16**, 819-825 (2017).

3. Yu, J. et al. Electrically control amplified spontaneous emission in colloidal quantum dots. *Sci. Adv*. **5**, eaav3140 (2019).
